# Single-Port Robot-Assisted Post-Chemotherapy Unilateral Retroperitoneal Lymph Node Dissection: Feasibility and Surgical Considerations

**DOI:** 10.1590/S1677-5538.IBJU.2025.0091

**Published:** 2025-03-20

**Authors:** Sisto Perdonà, Alessandro Izzo, Roberto Contieri, Francesco Passaro, Savio Domenico Pandolfo, Roberto Corrado, Giovanna Canfora, Rocco Damiano, Riccardo Autorino, Gianluca Spena

**Affiliations:** 1 Istituto Nazionale Tumori di Napoli Department of Urology Napoli Italy Department of Urology, Istituto Nazionale Tumori di Napoli, IRCCS, Fondazione "G. Pascale", Napoli, Italy; 2 Federico II University of Naples Department of Neurosciences and Reproductive Sciences and Odontostomatology Naples Italy Department of Neurosciences and Reproductive Sciences and Odontostomatology, Federico II University of Naples, Naples, Italy; 3 Istituto Nazionale Tumori di Napoli Department of Anesthesiology Napoli Italy Department of Anesthesiology, Istituto Nazionale Tumori di Napoli, IRCCS, Fondazione "G. Pascale" Napoli, Italy; 4 Magna Graecia University of Catanzaro Department of Urology Catanzaro Italy Department of Urology, Magna Graecia University of Catanzaro, Catanzaro, Italy; 5 Rush University Medical Center Department of Urology Chicago IL USA Department of Urology, Rush University Medical Center, Chicago, IL, USA

## Abstract

**Introduction:**

Retroperitoneal lymph node dissection (RPLND) is indicated for testicular cancer patients with residual masses post-chemotherapy or stage I-II non-seminomatous germ cell tumors (NSGCT) (1, 2). Open RPLND remains the standard but carries significant morbidity. The laparoscopic approach, while minimally invasive, presents notable technical challenges (3). Robotic-assisted RPLND (rRPLND) offers a minimally invasive alternative with comparable oncological outcomes (4, 5). The Da Vinci Single Port (SP) system presents new possibilities for reducing surgical morbidity (6, 7).

**Methods:**

We report a case of SP-rRPLND using a unilateral modified template and a lower anterior access (LAA) in a 41-year-old man with NSGCT (pT2, UICC Stage IB) who underwent left orchiectomy, followed by adjuvant chemotherapy. A CT scan revealed a 3.5 cm residual retroperitoneal mass in the left hilar region.

The surgical procedure, performed with the Da Vinci SP system, involved a 2.5 cm McBurney incision for retroperitoneal access. Instrument configuration followed a "Camera below" setting. The unilateral left-sided modified template guided dissection from the aortic bifurcation to the renal hilum, preserving vascular structures. A 3,5 cm residual mass and para-aortic nodes were excised with the help of flexible Greena® applicator for clips.

**Results:**

Anesthetic management prioritized opioid-sparing techniques to enhance recovery. The patient received regional anesthesia, multimodal analgesia, and had an NRS pain score of 0 at discharge.

The console time was 79 minutes, with minimal blood loss and no complications. The patient resumed oral intake on postoperative day 1 and was discharged on day 2. Postoperative recovery was uneventful, with no complications or need for conversion to open or laparoscopic surgery.

Final histopathological examination revealed a germ cell tumor with features suggestive of immature teratoma, along with over 10 lymph nodes showing sinus histiocytosis. At six months post-RPLND, the patient remains disease-free, with a good general condition and no new symptoms. Tumor markers (AFP, β-hCG, LDH) are within normal limits, and CT imaging shows ***no evidence*** of recurrence or residual retroperitoneal masses. Renal function and hormonal profile are stable. Given prior chemotherapy exposure, cardiovascular monitoring is advised. Follow-up will continue with clinical exams and tumor markers every 3-4 months, with the next CT scan planned at 12 months, unless symptoms warrant earlier imaging.

**Conclusions:**

As far as we know this is the first reported case of SP-rRPLND in Europe. The LAA provides safe access while minimizing morbidity, potentially improving recovery (8). A unilateral approach, avoiding transperitoneal access, may further reduce morbidity (9). Future studies should validate long-term oncological outcomes and compare SP-rRPLND with multiport and open approaches. SP-rRPLND represents a promising advancement in minimally invasive testicular cancer surgery.

Available at: http://www.intbrazjurol.com.br/video-section/20250091_Spena_et_al

**Video 1:** 

## Data Availability

https://zenodo.org/records/14841220

## References

[1] Tufano A, Cilio S, Spena G, Izzo A, Castaldo L, Grimaldi G (2024). Unilateral Post-Chemotherapy Robot-Assisted Retroperitoneal Lymph Node Dissection for Stage II Non-Seminomatous Germ Cell Tumors: Sexual and Reproductive Outcomes. Cancers (Basel).

[2] Melão BVLA, de Amorim LGCR, Sanches MR, Gomes GV, Gewehr DM, Moreira LHO (2024). Primary Retroperitoneal Lymph Node Dissection for Clinical Stage II A/B Seminomas: A Systematic Review and Meta-Analysis. Int Braz J Urol.

[3] Santos VE, Fornazieri L, Brazão ES, Pinto PR, da Costa WH, Zequi SC (2023). Primary laparoscopic RPLND for pure seminona metastasis: feasibility of supine and lateral approaches. Int Braz J Urol.

[4] Gomes DC, Da Costa WH, Brazão ÉS, Vergamini LB, Ricci BV, Zequi SC (2021). Robot-assisted retroperitoneal lymphadenectomy (RPLND): video case report. Int Braz J Urol.

[5] Ghoreifi A, Sheybaee Moghaddam F, Mitra AP, Khanna A, Singh A, Chavarriaga J (2024). Oncological Outcomes Following Robotic Postchemotherapy Retroperitoneal Lymph Node Dissection for Testicular Cancer: A Worldwide Multicenter Study. Eur Urol Focus.

[6] Ditonno F, Franco A, Licari LC, Bologna E, Manfredi C, Katz DO (2024). Implementation of single-port robotic urologic surgery: experience at a large academic center. J Robot Surg.

[7] Perdonà S, Izzo A, Tufano A, Passaro F, Quarto G, Aveta A (2025). Advancing Surgical Management of Penile Cancer: Single Port Bilateral Inguinal Lymph Node Dissection. Int Braz J Urol.

[8] Pellegrino AA, Chen G, Morgantini L, Calvo RS, Crivellaro S (2023). Simplifying Retroperitoneal Robotic Single-port Surgery: Novel Supine Anterior Retroperitoneal Access. Eur Urol.

[9] Masterson TA, Cary C (2018). The Use of Modified Templates in Early and Advanced Stage Nonseminomatous Germ Cell Tumor. Adv Urol.

